# Poisoning by Stramonium

**Published:** 1850-11

**Authors:** Ira E. Oatman

**Affiliations:** Dundee, Ill.


### ARTICLE IX.

*Poisoning by Stramonium.*—By Ira E. Oatman, M.D., of Dundee, Ill.

On the 21st of Sept., at 7 P. M., I was called in haste to see a little boy, 3 or 4 years old. He presented the following symptoms, viz.: Hot skin, flushed countenance, full pulse, dilated pupils and total blindness. He had occasional convulsions, violent spasms, and furious delirium,—greatly increased by giving liquids or medicines, or by putting him into warm water. He screamed wildly and hideously; and raved and yelled equal to the worst case of delirium tremens. The earlier symptoms were those of poisoning with alcohol: but his brother thought he had eaten a thorn apple; and he said, (when first taken) yes, he had "eat it all up." Some ground mustard advised by another physician had not vomited him, and his agony was intense. The Sul. Zinc. and Ipecac., in large emetic doses, assisted by copious draughts of warm water, dislodged the seeds and inner portions of the pod with difficulty from the stomach. He then sank into a profound sleep, with dilated and immovable pupils and general insensibility. I

ordered ½ oz. Castor Oil, in warm milk, every hour, and the same, per enemata. Gave some stimulants, and applied cold to the head. Early the next morning I was awakened by the father of the child, who said he had come to tell me that I need not call again, for his boy was "well." He is now in good health.

I submit this case that every one may be warned against this poison, so abundant in all parts of the country.

